# Promise, progress, and problems in whole disc tissue engineering

**DOI:** 10.1002/jsp2.1015

**Published:** 2018-05-23

**Authors:** Sarah E. Gullbrand, Lachlan J. Smith, Harvey E. Smith, Robert L. Mauck

**Affiliations:** ^1^ Translational Musculoskeletal Research Center Corporal Michael J. Crescenz VA Medical Center Philadelphia Pennsylvania; ^2^ McKay Orthopaedic Research Laboratory, Department of Orthopaedic Surgery University of Pennsylvania Philadelphia Pennsylvania; ^3^ Department of Neurosurgery University of Pennsylvania Philadelphia Pennsylvania; ^4^ Department of Bioengineering University of Pennsylvania Philadelphia Pennsylvania

**Keywords:** animal models, biomaterials, biomechanics, disc degeneration, mesenchymal stem cells

## Abstract

Intervertebral disc degeneration is frequently implicated as a cause of back and neck pain, which are pervasive musculoskeletal complaints in modern society. For the treatment of end stage disc degeneration, replacement of the disc with a viable, tissue‐engineered construct that mimics native disc structure and function is a promising alternative to fusion or mechanical arthroplasty techniques. Substantial progress has been made in the field of whole disc tissue engineering over the past decade, with a variety of innovative designs characterized both in vitro and in vivo in animal models. However, significant barriers to clinical translation remain, including construct size, cell source, culture technique, and the identification of appropriate animal models for preclinical evaluation. Here we review the clinical need for disc tissue engineering, the current state of the field, and the outstanding challenges that will need to be addressed by future work in this area.

## 1 INTRODUCTION

Back and neck pain place a significant social and economic burden on modern society, affecting nearly 1 in 2 individuals annually. In the United States alone, these conditions are associated with an estimated $194 billion in yearly medical costs and lost wages.^1^ Back pain is commonly associated with degenerative disc disease, a progressive condition with complex underlying etiology, during which component tissues undergo an array of cellular and structural changes that ultimately compromises biomechanical function. Although the pathological manifestations of disc degeneration are well characterized, the underlying causes and the origins of discogenic pain are still not well understood, impeding the development of effective therapies. Current treatments for disc degeneration do not restore native disc structure and function, and thus exhibit limited long‐term efficacy. Tissue engineering offers the promise of generating de novo, living structures that recapitulate the form, function, and biology of healthy host tissues with the potential to treat a variety musculoskeletal disorders, including disc degeneration. Here, we review recent progress in the field of whole disc tissue engineering as well as the barriers that will need to be addressed for the successful clinical translation of engineered discs.

## 2 INTERVERTEBRAL DISC STRUCTURE AND MECHANICAL FUNCTION

The intervertebral discs of the spine are composite structures composed of the central nucleus pulposus (NP), the peripheral annulus fibrosus (AF), and the superior and inferior cartilaginous end plates. The extracellular matrix (ECM) of the NP is composed primarily of proteoglycans and type II collagen; the high proteoglycan content of healthy NP tissue results in high water content.^2^, ^3^ The AF ECM is composed primarily of type I and II collagen fibers oriented at alternating angles of approximately ±30° relative to the long axis of the spine, organized in roughly concentric lamellar layers.^4^, ^5^ This hierarchical structure is crucial to the mechanical function of the disc, which is to bear and distribute loads while simultaneously permitting motion. When subjected to compression during normal daily activity, hydrostatic pressure develops in the NP, placing the annulus in tension, thus facilitating the support of axial loading.^6^, ^7^

Between the intervertebral disc and the adjacent vertebral bodies are the cartilage endplates (EP), thin layers of hyaline‐like cartilage composed of proteoglycans and type II and IX collagens.^8^ The low permeability of these EP relative to the porous underlying bone limits fluid diffusion and enables the NP to pressurize. As the intervertebral discs are the largest avascular structures in the body, the EP also play a critical role in the health of disc cells. Nearly all nutrients received by disc cells must diffuse from vascular buds in the adjacent vertebrae, through the cartilage EP, and into the disc.^9^, ^10^ As such disc tissue is generally hypoxic under physiologic conditions, with oxygen tensions of ~2 to 5%.^11^ Glucose availability decreases and lactic acid concentrations increase with increasing distance from this vasculature supply.^12^ The permeability, composition, and vascularity of the EP significantly impact diffusion of small molecules into the disc.^13^, ^14^ In addition to this challenging nutritional environment, disc cells are also subject to large magnitudes of loading in both compression and torsion during spinal motion associated with normal daily activity. Loads on the intervertebral disc can exceed up to 5 times body weight, depending on nature of the activity.^15^

Within the context of, and in part due to, this harsh physical environment, disc cellularity is very low—averaging 9000 cells/mm^3^ in the AF and 4000 cells/mm^3^ in the NP.^16^ Cells within the AF are fibrochondrocyte‐like in nature, while the cells of the NP are more chondrocyte‐like.^17^, ^18^ NP cells arise from the notochord during development, and are uniquely suited to survival in the low‐nutrient environment of the NP via their stable expression of hypoxia inducible factor (HIF‐1α), which promotes the transcription of glycolytic enzymes that facilitate anaerobic metabolism.^19^, ^20^, ^21^

## 3 INTERVERTEBRAL DISC DEGENERATION AND TREATMENT

Degeneration of the intervertebral discs may occur with aging or following injury. While the exact pathophysiology of disc degeneration remains unclear, it is known to involve a progressive cascade of cellular, compositional, and structural changes. The earliest degenerative changes to the disc typically manifest in the NP as a slow loss of proteoglycans, which results in a decrease in osmotic pressure, a loss of hydration and a reduced capacity of the disc to carry compressive loads.^22^, ^23^ Degenerative discs therefore lose fluid and height more rapidly, leading to disc bulging.^22^ Alterations in disc ECM with degeneration are mediated in part by increased production of pro‐inflammatory cytokines such as interleukin‐1β and tumor necrosis factor α, which activate a variety of downstream catabolic molecules and downregulate the expression of ECM genes, leading to a disruption of disc matrix homeostasis.^24^, ^25^ Later stages of degeneration are characterized by fibrosis of the NP, cell death, disorganization and infolding of the annulus layers, and a collapse in disc height.^23^, ^26^

Disc degeneration is frequently associated with axial spine pain and neurogenic extremity pain.^27^ There are multiple potential pain generators in the spine, including the intervertebral disc itself (discogenic pain) and the surrounding boney structures (the facet joints and vertebral endplate), as well as compression of the adjacent neural structures due to loss of disc height, bulging or herniation.^28^ While its origins are still not fully understood, spine pain places a significant social and economic burden on modern society. Back pain is the number one contributor to years lived with disability for adults in the United States.^29^ The economic burden of back pain is also significant, as it is associated with an estimated $190 billion in medical costs and lost wages each year in the United States.^27^

The first line of clinical treatment for pain associated with disc degeneration is generally nonoperative, and may include physical therapy, nonsteroidal antiinflammatory drugs, muscle relaxers, aerobic conditioning, traction and chiropractic manipulation, and epidural steroid injections.^30^ For end‐stage disc degeneration and debilitating axial back pain that is unresponsive to conservative treatment, the current surgical option is fusion of the spinal motion segment. The fusion procedure removes the degenerative disc and immobilizes the vertebra of the involved interspace in an effort to relieve pain by eliminating motion and decompressing the neural elements from the restoration of disc and foraminal height. However, spinal fusion does not restore native spinal motion segment structure or mechanical function. The reduction in range of motion at the fused level also contributes to hypermobility of adjacent spinal levels, which may accelerate degeneration of the associated discs.^31^ Furthermore, pain relief only occurs in 60 to 80% of spinal fusion patients, and some meta‐analyses of randomized, controlled trials have suggested no clinically significant difference in outcomes following fusion compared to conservative treatment.^32^, ^33^, ^34^

Total disc arthroplasty, which removes the degenerative disc and replaces it with a mechanical articulating device, is an alternative to spinal fusion, with the goal of preserving spinal motion and so potentially decreasing the risk of adjacent segment degeneration.^35^ Despite these theoretical benefits, systematic reviews have shown no clinically significant benefit of mechanical disc arthroplasty over fusion in either the cervical or lumbar spine.^35^, ^36^, ^37^ The reason for this lack of efficacy remains unclear, but may be related to the limits of mechanical arthroplasty devices to fully recapitulate the structure, kinematics, and mechanical function of the native disc. Concerns have also emerged regarding the subsidence and potential for wear particle production of these mechanical arthroplasty devices,^38^ and revision of a failed mechanical arthroplasty can be challenging with a high incidence of complications.

Considering the limitations of current treatment options, there is a significant need to develop new therapeutics for disc degeneration. Replacement of a degenerate disc with a biologic, tissue‐engineered construct is a promising potential treatment strategy. To succeed, a tissue‐engineered disc would need to integrate with the adjacent native tissues, and mature and remodel in response to the in vivo environment, thereby restoring the healthy structure and mechanical function of the motion segment.

## 4 PROGRESS IN WHOLE DISC TISSUE ENGINEERING

To address the concept of disc replacement, the field of whole disc tissue engineering has progressed markedly over the past decade, from the first in vitro characterizations of composite tissue engineered discs in 2004 to the implantation of entire, living, engineered discs in a large animal model in 2017 (Table 1). The following sections will detail the progress in biomaterial selection and fabrication, cell and tissue culture methods, and in vivo models that have advanced this field.

**Table 1:** Summary table of studies to date with a primary focus on whole intervertebral disc tissue engineering

	AF composition	NP composition	Other components	Dimensions	Cell type used	Maturation strategy	In vivo evaluation	Outcomes measures
Mizuno et al, 2004, 2006^40^, ^41^	Non‐woven polyglycolic acid mesh	Alginate	n/a	2 mm high, 10 mm diameter	Ovine NP and AF cells	Subcutaneous implantation for up to 16 weeks	n/a	Histology, biochemistry, compressive mechanics
Nesti et al, 2008^45^	Electrospun poly(l‐lactic acid)	Hyaluronic acid	n/a	1 cm^2^	Human MSCs	Chemically defined media + TGF‐β1 for 28 days	n/a	Histology, biochemistry, PCR
Nerurkar et al, 2010^49^	Electrospun poly (ε‐caprolactone)	2% agarose	n/a	3 mm high, 10 mm diameter	Bovine MSCs	Chemically defined media + TGF‐β3 for 6 weeks	n/a	Histology, biochemistry, compressive mechanics
Bowles et al, 2011^42,^	Collagen	Alginate	n/a	2 mm high, 4 mm diameter	Ovine NP and AF cells	Serum containing media with ascorbic acid for 2 weeks	Rat tail disc space for 6 months	Histology, disc height, MRI, motion segment mechanics, biochemistry
Zhuang et al, 2011^43^	Demineralized bone matrix gelatin	Collagen‐II/ hyaluronate/ chondroitin‐6‐sulfate	n/a	n/a	Lupine NP and AF cells	Subcutaneous implantation for up to 12 weeks	n/a	Histology, biochemistry
Lazebnik et al, 2011^47^	Electrospun poly (ε‐caprolactone)	2% agarose	n/a	2 mm high, 8 mm diameter	Porcine articular chondrocytes	n/a	n/a	Cell viability, compressive mechanics, histology
Bowles et al, 2012^50^	Collagen	Alginate	n/a	1 mm high, 3.23 mm anterior‐poster width, 3.8 mm lateral width	Ovine AF and NP cells	Serum containing media with ascorbic acid for 2 weeks	Rat lumbar disc space for 16 weeks	Histology, disc height
Park et al, 2012^44^	Silk fibroin	Fibrin/hyaluronic acid	n/a	3 mm high, 8 mm diameter	Porcine articular chondrocytes and AF cells	Chemically defined media + TGF‐β1 for 4 weeks	n/a	Histology, cell viability, biochemistry, qPCR
Chik et al, 2014^52^	Collagen	Collagen/GAG Co‐precipitate	Osteochondral bi‐layer of collagen and MSCs	10 mm high, 10 mm diameter	Lupine MSCs	Chemically defined media + TGF‐β1 for 21 days	n/a	Histology, cell viability, torsional mechanics
Xu et al, 2015^59^	Decellularized bone matrix	Articular cartilage ECM	n/a	3 mm high, 10 mm diameter	Porcine NP and AF cells	n/a	Subcutaneous implantation for 6 weeks	Histology, cell viability, qPCR, compressive mechanics
Hudson et al, 2015^57^	Collagen	Alginate	n/a	2 mm high, 4 mm diameter	Ovine NP and AF cells	Serum containing media with ascorbic acid +1 Hz compressive loading for 2 weeks	n/a	Histology, biochemistry, compressive mechanics
Hudson et al, 2015^57^	Collagen	Alginate	n/a	2 mm high, 4 mm diameter	Human MSCs	Serum containing media with ascorbic acid + hypoxia (2% O_2_) for 4 weeks	n/a	Histology, biochemistry, compressive mechanics
Martin et al, 2017^51^	Electrospun poly (ε‐caprolactone)	Methacrylated hyaluronic acid	n/a	2 mm high, 5 mm diameter	Bovine NP and AF cells	Serum containing media + ascorbic acid and TGF‐β3 and OR chemically defined media + TGF‐β3	Subcutaneous implantation for 5 weeks	Histology, MRI T2 mapping, biochemistry, cell metabolic activity
Iu et al, 2017^48^, ^66^, ^67^	Electrospun polycarbonate urethane	Scaffold free	Calcium polyphosphate endplate	5 mm high, 10 mm diameter	Bovine NP and AF cells	Serum containing media with ascorbic acid for 2 weeks	Calf tail disc space for 4 weeks	Histology, biochemistry, interfacial shear strength
Moriguchi et al, 2017^58^	Collagen	Alginate	n/a	3 mm high, 10 mm diameter	Canine NP and AF cells	Serum containing media with ascorbic acid for 2 weeks	Canine cervical disc space for 16 weeks	Histology, MRI T2 mapping, disc height
Martin et al, 2017^51^	Electrospun poly (ε‐caprolactone)	Methacrylated hyaluronic acid	Poly (ε‐caprolactone) foam endplate	2–5 mm high, 5 mm diameter	Bovine NP and AF cells	Chemically defined media + TGF‐β3 for 5 weeks	Rat tail disc space for 5 weeks	Histology, biochemistry, compressive mechanics, MRI T2 mapping, μCT

### 4.1 Biomaterial selection for whole disc tissue engineering

**Figure 1:**
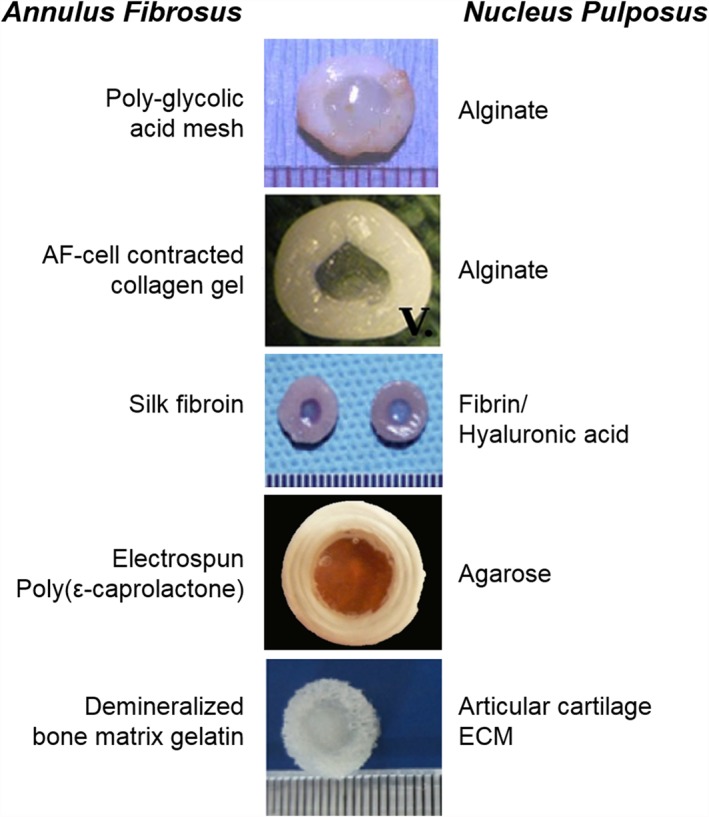
Examples of select tissue‐engineered discs (figures reprinted with permissions from References 39, 41, 44, 49, 59), and a listing of the biomaterials used in fabricating the AF and NP regions of each design

In general, whole disc tissue engineering involves combining cells with biomaterial scaffolds to generate a composite structure with distinct NP and AF regions (Figure 1).^39^ Engineered AF tissues have previously been fabricated en bloc from porous materials including polyglycolic acid, demineralized bone matrix gelatin, collagen, and silk fibroin, mimicking basic geometry but omitting microarchitectural detail.^40^, ^41^, ^42^, ^43^, ^44^ To better recapitulate native AF architecture, other strategies have included the use of layers of nanofibrous electrospun polymers, including poly(ε‐caprolactone) (PCL), polycarbonate urethane, and poly lactic‐co‐glycolic acid.^45^, ^46^, ^47^, ^48^ These materials better mimic the structure of the native disc, from the alignment of collagen fibers within a single fibrous lamella to the angle‐ply nature of the AF as a whole. The NP region of composite‐engineered discs is commonly fabricated from a hydrogel, to replicate the hydrated, gel‐like composition of the native tissue. Various hydrogels have been used, including alginate, agarose, and hyaluronic acid.^41^, ^47^, ^49^, ^50^, ^51^ Others have utilized ECM‐derived materials for NP tissue engineering, including collagen and Glycosaminoglycan (GAG) co‐precipitates, and mixtures of collagen II, hyaluronate and chondroitin‐6‐sulfate.^43^, ^52^ Several groups have also included analogs for the adjacent vertebral EP fabricated from porous calcium polyphosphate, collagen gels, and porous PCL.^48^, ^52^, ^53^, ^54^

### 4.2 Cell sources and culture strategies for whole disc tissue engineering

The cell types utilized in conjunction with these biomaterials to create viable constructs have included native AF and NP cells, mesenchymal stem cells (MSCs), and articular cartilage chondrocytes. Acellular‐engineered discs have also been implanted in the disc spaces of small animal models; however, the ideal balance of ECM components has generally not been achieved, as matrix deposition by infiltrating host cells is largely collagenous and did not match that of the native tissue.^46^, ^55^ Seeding an engineered disc with cells in vitro*,* and preculturing to differentiate those cells and accelerate matrix deposition and maturation, facilitates better recapitulation of native disc composition and mechanical properties. However, divergent approaches exist regarding the most appropriate manner in which to culminate in cell‐seeded engineered discs that are mature and mechanically functional in the in vivo environment. Two main strategies have been pursued—maturation of the construct in vitro via pre‐culture prior to in vivo implantation,^42^, ^44^, ^45^, ^49^, ^51^, ^52^, ^53^, ^54^, ^56^, ^57^, ^58^ and implantation of an immature construct without preculture that subsequently undergoes maturation in the in vivo space.^40^, ^41^, ^43^, ^59^

The first composite‐engineered discs implanted into a living animal were composed of an ovine AF cell‐seeded polyglycolic acid mesh as an analog for the AF region, and alginate seeded with ovine NP cells for the NP region.^40^ These constructs were cultured for 1 day prior to subcutaneous implantation in athymic rats, where they continued to mature for up to 16 weeks. These engineered discs matured compositionally in the in vivo space, with increases in GAG, collagen, and DNA content that correlated to increases in compressive mechanical properties.^40^, ^41^ However, biochemical content of these constructs did not reach the levels of the native ovine disc, with the exception of NP proteoglycan content, and the magnitude of compressive modulus achieved (50 kPa) remained below the axial compressive modulus of a spinal motion segment (3 to 10 MPa). Similar increases in matrix content following subcutaneous implantation were noted in more recent studies with disc cells seeded into engineered constructs fabricated using alternative scaffolds.^43^, ^59^ Although evidence of integration between the AF and NP regions of these constructs was noted histologically following in vivo implantation, the mechanical properties of these interfaces were not characterized. Additionally, it was unclear from these studies whether the matrix accumulation observed in these constructs was due to the seeded cells or was produced by infiltrating host cells.

While this work illustrated that engineered discs can functionally mature in an in vivo environment, the subcutaneous space provides a wide variety of biological signals to an engineered construct that may not necessarily be conducive to the production of disc‐like matrix. Relying on subcutaneous implantation for engineered disc maturation is also undesirable from a clinical translation perspective. Although it is currently unclear what maturation state an engineered disc should reach in order to achieve native tissue equivalence in vivo within the spine, it is likely that some degree of functional maturation will be necessary. Therefore, several groups have utilized various in vitro culture strategies to stimulate disc‐like matrix deposition and enhance the mechanical function of engineered disc constructs prior to in vivo implantation.^42^, ^48^, ^51^, ^54^, ^58^

Most commonly, engineered discs have been cultured in a chemically defined media containing multiple factors to stimulate ECM production, including TGF‐β, ascorbate‐2‐phosphate, proline, and dexamethasone. This media formulation was originally designed to induce chondrogenesis of MSCs, and has been extensively used to promote matrix production in a variety of engineered musculoskeletal tissues, including cartilage and meniscus.^60^, ^61^, ^62^, ^63^ Robust matrix production has also been observed in both disc cell‐seeded and MSC‐seeded engineered discs cultured in chemically defined media supplemented with TGF‐β, with construct matrix levels exceeding those achieved via subcutaneous implantation alone.^44^, ^45^, ^49^, ^52^, ^54^ The robust matrix deposition generated by culture in chondrogenic media also results in improvements in the compressive and viscoelastic mechanical properties of engineered discs, although native levels with respect to these properties have not yet been achieved.^49^, ^54^ Furthermore, compared to alternative media formulations that include serum, chemically defined media with TGF‐β3 promoted not only the greatest deposition of GAG and collagen in engineered discs in vitro, but also enhanced matrix retention and metabolic activity following in vivo implantation.^51^

Other recent studies have investigated the effects of alternative exogenous stimuli, including hypoxia and dynamic compressive loading, on engineered disc maturation in vitro.^56^, ^57^ In these studies, engineered discs were cultured in a serum containing media without TGF‐β, and were composed of either MSC or disc cell‐seeded alginate hydrogels for the NP region, combined with collagen gels for the AF region that self‐assembled in culture to become circumferentially aligned. Dynamic compression across a range of strain magnitudes over 2 weeks of culture led to dose‐dependent increases in AF and NP cell‐seeded engineered disc GAG and collagen contents, accompanied by improvements in the instantaneous and equilibrium moduli. These increases due to mechanical stimulation were 2‐ to 4‐fold higher than free swelling controls, though mechanical and biochemical parameters still remained below native values.^57^ For the fabrication of engineered discs with human MSCs, monolayer expansion in hypoxia (5% O_2_) prior to seeding into the 3D construct improved compressive mechanical properties compared to normoxic expansion (21% O_2_). This may have been due to improved integration between the AF and NP components of the engineered discs, as there was little effect of hypoxic culture on construct biochemistry in the individual compartments.^56^ Interestingly, the mechanical properties and biochemical content of disc cell‐seeded constructs and human MSC‐seeded constructs in the aforementioned studies fell within a similar range. This suggests that, under culture conditions free of TGF‐β, disc cells and MSCs can perform similarly as cell sources for disc tissue engineering; however, the beneficial effects of hypoxic culture and dynamic loading may require additional optimization or longer applications to realize their potential.

### 4.3 In vivo evaluation of engineered discs in the spine

While various designs of composite tissue‐engineered discs have been described in the literature and characterized in vitro, there have been relatively few studies that have assessed the performance of a tissue‐engineered disc upon in vivo implantation within the disc space. The first total disc replacement with a tissue‐engineered construct was performed in the rat caudal spine, with a construct consisting of an NP cell‐seeded alginate hydrogel and an AF cell‐contracted collagen gel.^42^ These engineered discs were cultured for 2 weeks in serum‐containing media prior to implantation to allow the AF cells to remodel the collagen gel to generate circumferential alignment in this disc region.^64^ Six months after implantation in the rat caudal spine, disc height was maintained and motion segment mechanical properties were similar to that of the native rat caudal disc. The implanted engineered discs produced new matrix in vivo, such that AF and NP proteoglycan and collagen levels reached that of the native rat tail disc.^42^ After 8 months in vivo, Magnetic Resonance Imaging (MRI) T2 mapping demonstrated that NP T2 values within these tissue‐engineered discs remained at native levels, although T1ρ mapping and histology indicated reductions in proteoglycan content of the engineered compared to native discs, which was accompanied by a loss of disc height and NP size.^65^

Engineered discs of the same design have recently been translated from the rat tail model into a canine cervical disc replacement model, the first study to report the implantation of a tissue‐engineered disc in a large animal model. In contrast to the rat tail implantation studies, where the engineered discs remained in place without the need for fixation, in this larger canine model, displacement of the construct occurred in half of the animals investigated.^58^ In successfully retained constructs, however, disc height remained significantly higher than discectomy controls after 16 weeks, and evidence of construct integration with the native tissue was observed via histological analysis. These findings are similar to the results of implantation of engineered discs of the same design within the rat lumbar spine.^50^ In the canine cervical spine, proteoglycan content within the engineered disc appeared to decrease with increasing duration of implantation, and MRI T2 relaxation times in the NP were not significantly different from discectomy after 16 weeks.^58^

Alternate designs for engineered discs have also been evaluated in the rat caudal spine. In these studies, tissue‐engineered disc‐like angle ply structures (DAPS), seeded with AF and NP cells, were precultured in chemically defined media with TGF‐β3 for 5 or 10 weeks prior to implantation in the rat caudal spine.^54^ In contrast to the previous rat caudal implantation study, the DAPS‐implanted motion segment was immobilized via a ring type external fixator, which was necessary to prevent expulsion of the DAPS from the disc space.^46^ These differing needs for fixation in the rat caudal spine may be due to differences in the preimplantation stiffness of these 2 designs of engineered discs—with stiffer, more mature constructs perhaps necessitating fixation compared to less mature engineered discs. After 5 weeks in vivo, although the toe and linear region moduli of the DAPS‐implanted motion segments were not significantly different from the native rat tail, the NP region of the DAPS exhibited a progressive loss of proteoglycan content, and there was little evidence of integration of the DAPS with adjacent native tissue structures. In more recent studies, apposition of acellular PCL foam EP to the DAPS substantially improved their in vivo performance, resulting in retention of MRI T2 signal and proteoglycan content, and histological and μCT evidence of integration of the EP with the adjacent vertebral bone.^54^

Inclusion of an endplate region to stimulate integration of tissue‐engineered discs in vitro and in vivo has also been attempted in constructs composed of layered electrospun polycarbonate urethane for the AF region, surrounding a calcium polyphosphate cylinder seeded directly with NP cells.^53^, ^66^ These constructs were cultured for 2 weeks in serum‐containing media prior to implantation in a defect created in the bovine caudal disc space and adjacent vertebral body for 4 weeks.^48^ While there was histological evidence of integration between the engineered disc components after in vivo implantation, these constructs were not sized to replace the whole bovine caudal disc, and there was evidence of subsidence into the vertebral body.^48^

## 5 CHALLENGES FOR in vivo TRANSLATION OF AN ENGINEERED DISC

Despite the marked progress in whole disc tissue engineering over the past decade, there are many challenges that will need to be overcome before clinical translation can be achieved. In this section, we outline a number of these challenges and progress toward their resolution.

### 5.1 Scale up of tissue‐engineered discs to clinically relevant sizes

**Figure 2:**
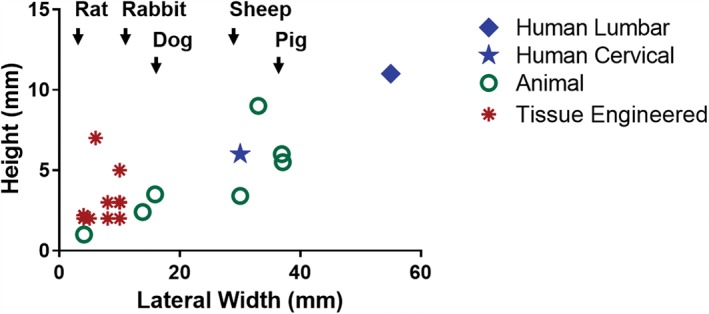
Plot of the height and lateral width of tissue engineered discs reported in the literature compared to the average height and lateral width of human cervical and lumbar discs, and the discs of animal models commonly used for spine research

One of the first challenge for disc tissue engineering is generating constructs of human size and geometry. While it is often possible to engineer small‐scale tissues that exhibit properties equivalent to native tissue, scale up to human dimensions reveals limitations in culture methods. For tissue‐engineered discs reported in the literature, constructs produced to date have not reached dimensions matching those of either human cervical or lumbar intervertebral discs (Figure 2). The average size of engineered discs is 3.1 mm in height and 7.5 mm in width, while the human lumbar disc has an average height of 11 mm and average widths in the anterior‐posterior and lateral directions of 37 mm and 55 mm, respectively.^68^ The human cervical spine may be a more realistic target for a tissue‐engineered disc replacement—not only are the mechanical demands lesser in the cervical spine, but the disc dimensions are closer to the current dimensions of engineered constructs, with a height of 5 to 6 mm and average widths in the anterior‐posterior and lateral directions of 20 mm and 30 mm, respectively.^69^, ^70^ Even so, this would represent a 2‐fold increase in both the height and width compared to current engineered disc constructs.

**Figure 3:**
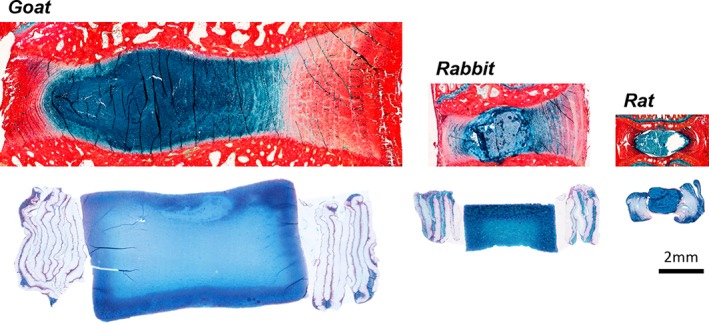
Sagittal Alcian blue (glycosaminoglycans) and picrosirius red (collagens) stained histology sections from rat, rabbit, and goat intervertebral discs, and tissue‐engineered discs at matching size scales that were cultured in chemically defined media with TGF‐β3 for 5 weeks

The culture and maturation of large engineered constructs also poses significant challenges, as diffusional constraints of media components can result in heterogeneity in cell viability and matrix distribution from the periphery to the center of the construct.^62^, ^71^, ^72^, ^73^ This has been well documented in the cartilage tissue engineering literature, where substantial differences in mechanical function across the construct depth have been reported.^74^, ^75^ In our work on engineered discs, we noted significant heterogeneity in matrix distribution and loss of cell viability in the center of constructs that were scaled up from a size appropriate for use in the rat caudal spine (2 mm high, 5 mm diameter) to a size comparable to the goat and human cervical disc space (6 mm high, 20 mm diameter) (Figure 3).^76^ A number of strategies could be implemented to enhance transport into these large‐size engineered discs in culture, including the inclusion of nutrient channels through the construct depth, the use of alternate media formulations that promote tissue formation through the depth (such as the use of latent TGF‐β as a component of chondrogenic media), and culture in bioreactors that are designed to promote the convective transport of vital nutrients into these larger constructs via cyclic mechanical loading.^57^, ^74^, ^75^, ^77^, ^78^ Many of these techniques have been applied to cartilage tissue engineering, and thus lessons learned from that field may expedite the successful generation of large‐scale engineered discs. However, due to the myriad of differences between articular cartilage and disc, many of the aforementioned strategies will likely require additional optimization for application to disc tissue engineering.

Furthermore, even if the maturation of human‐sized engineered discs can be achieved in culture where nutrients are available within the culture media at super‐physiological levels, engineered discs will face further nutritional challenges in the in vivo environment.^12^, ^79^ This may be exacerbated by the fact that tissue‐engineered discs are usually seeded at cell densities several‐fold higher than the cell density of the native tissue in order to speed functional maturation of the construct during in vitro culture.^76^ It is possible that a certain amount of cell death following in vivo implantation will be acceptable, so long as the formed matrix is retained and the remaining cell population is sufficient to maintain long‐term homeostasis of the tissue overall. Strategies to enhance nutrient transport into engineered discs in vivo, such as enhancement of the vertebral endplate vasculature or stimulation of convective transport via dynamic loading, may ultimately be necessary for the success of engineered constructs in vivo.^14^, ^80^

As another approach, acclimation of engineered discs to the harsh low glucose and oxygen environment of the native disc may also improve in vivo performance, and could potentially be achieved via preculture methods to reduce construct metabolic activity prior to implantation. This could include mild hypothermia, serum starvation, hypoxic culture or other strategies that are applied for a period of time prior to implantation.^56^, ^81^, ^82^, ^83^ The design of engineered discs could also be modified to include material inclusions that provide the sustained release of small molecules (ie, IL‐1Ra, dexamethasone, glucose, TGF‐β) from within the construct to mitigate the inflammatory and nutrient‐poor environment of the degenerative spine and assist in maintaining engineered disc phenotype.^84^, ^85^, ^86^, ^87^

### 5.2 Cell sources

Identification of an optimal cell source for whole disc tissue engineering is another challenge limiting progress toward clinical translation. A variety of cell types have been utilized for disc tissue engineering, yet there is currently a lack of consensus on the optimal cell source. The majority of the work in the field has utilized AF and NP cells—a logical choice for initial in vitro and proof‐of‐concept studies as the native tissue cell type. The phenotype of healthy NP cells in particular has been thoroughly characterized, and a variety of scaffolds and hydrogels seeded with AF and NP cells have been shown to functionally mature with in vitro culture.^88^, ^89^, ^90^

Despite the rationale for using native disc cells for tissue engineering, there are substantial limitations to the utilization of this cell source for clinical translation. Obtaining autologous healthy human AF and NP cells would be challenging—removal of disc tissue from a patient's healthy disc is undesirable as the disc has an inherently poor repair capacity, and this may accelerate degeneration at that level. The use of allogeneic disc cells may provide a solution to this issue. Several studies in large animal models have demonstrated minimal immune reaction of allogeneic cells delivered to the disc space within either tissue‐engineered discs or hydrogel carriers, and a clinical trial of allogeneic MSCs for meniscal regeneration also demonstrated no significant immune response to allogeneic cell delivery.^48^, ^58^, ^91^, ^92^

Even if healthy human disc tissue could be obtained however, the cellularity of the disc is very low, and senescence of cells in the native disc is common, limiting their use in generating large, matrix‐rich‐engineered tissues.^16^, ^93^ Additionally, much of the work thus far with disc cells for tissue engineering has been achieved using cells from various animal species, where the donor animals are often juvenile.^43^, ^44^, ^47^, ^49^ Matrix production by cells derived from adult tissues for musculoskeletal tissue engineering is generally reduced compared to cells from juvenile tissues^94^, ^95^—thus, disc cells from adult human donors may not be as effective for disc tissue engineering. Other differentiated cell types such as articular chondrocytes have also been used in whole disc tissue engineering, but only for the NP region, and similar limitations exist in obtaining these cells with respect to donor site morbidity.^44^, ^47^ Nasal chondrocytes may be a promising alternate cell source for tissue engineering of the NP region, as it has been shown that they maintain their proliferative and matrix production capacity even in a simulated disc‐like microenvironment in vitro.^96^

As an alternative to native disc cells, autologous or allogeneic MSCs are an attractive cell source for disc tissue engineering. These cells can be easily obtained from bone marrow, adipose, or other tissues and have multi‐lineage potential.^97^, ^98^ TGF‐β can direct MSCs toward a disc‐like phenotype, and recent work has illustrated that the gene expression profile of TGF‐β differentiated MSCs is closer to native disc cells than articular chondrocytes.^60^, ^99^ In a direct comparison of MSCs and disc cells for whole disc tissue engineering, MSC‐seeded engineered discs sized for the rat caudal spine outperformed AF and NP cell‐seeded constructs with respect to proteoglycan and collagen production over 15 weeks of culture in chemically defined media supplemented with TGF‐β3.^54^ However, juvenile bovine cells were utilized in this study, so it is not yet clear how adult human MSCs would perform in comparison to disc cells. Additionally, MSCs are more sensitive than disc cells to nutrient deprivation, and therefore the challenges with scale up of engineered discs to larger sizes will likely be exacerbated in MSC seeded constructs.^62^, ^100^ Because of this, strategies to “precondition” MSCs to the harsh, environment of the disc may be necessary, such as preculture in hypoxia or low glucose media.^56^, ^101^

### 5.3 Benchmarks for success

Another challenge in translating whole disc tissue engineering to the clinic will be clearly defining the benchmarks that are necessary for an engineered disc to achieve during in vitro preculture to maximize success following in vivo implantation. The native disc has distinct biology, structure, composition, and mechanical properties, but it is important to examine the extent to which it is necessary to exactly replicate these characteristics in an engineered tissue prior to implantation. Ideally, a preculture strategy would yield a tissue‐engineered construct that matches the mechanical and biologic properties of the native disc. Mechanical benchmarks should encompass recapitulating not only native disc modulus and strain under physiologic multiaxial loading, but also matching the viscoelastic properties of the disc which are critical to its normal function in energy dissipation.^102^, ^103^ Biological benchmarks should include not only achieving native matrix distribution, but also recapitulating the phenotype and expression profile of native intervertebral disc cells. The phenotype of NP cells is well established,^20^ but definitive markers for AF cell phenotype have yet to be fully defined.^104^ Thus far, while the hierarchical structure of the native disc can be recreated by the biomaterials utilized for tissue engineering, the biology, composition, and mechanical properties of engineered discs have not yet achieved the levels of the native human disc. Additionally, no studies have characterized cell phenotype within whole engineered discs beyond the expression of matrix genes during culture or following in vivo implantation.

A wide variety of preculture strategies to promote engineered disc maturation have been described, yet there is no clear benefit of 1 culture strategy over another in terms of in vivo performance in the spine. Subcutaneous implantation studies comparing preculture media formulations suggest that the best in vivo performance, with respect to maintenance of cell viability and matrix content, was achieved by culturing engineered discs in chemically defined media with TGF‐β3.^51^ However, if we consider studies where engineered discs have been implanted in the rat caudal spine—relatively immature engineered discs with low initial mechanical properties and biochemical content performed well in the rat caudal disc space,^42^ but engineered discs of a different design matured in vitro prior to implantation lost proteoglycan matrix content upon in vivo implantation unless a polymer endplate region was included in the construct.^54^ Mechanical stimulation of engineered discs during preculture via bioreactor culture may be a promising strategy to drive the maturation of engineered discs to achieve native mechanical and biological benchmarks, as several previous studies have demonstrated that dynamic loading can enhance the functional properties of engineered cartilage or fibrocartilage.^78^, ^105^, ^106^ Both NP and AF cells are sensitive to mechanical stimuli, with low to moderate magnitudes and frequencies of loading generally eliciting an anabolic effect.^107^, ^108^, ^109^, ^110^ Recent work has also demonstrated that disc cells within whole engineered discs are responsive to dynamic compressive loading during culture^57^, ^111^; however, the optimal magnitudes, frequencies and durations of dynamic loading for the preculture of whole engineered discs still need to be established.

Ultimately, it may not be necessary to exactly match each individual characteristic of the native disc prior to implantation, so long as the overarching structure–function relationships (eg, positive correlations between matrix content and mechanical properties) in the engineered disc ultimately match that of the native tissue at some point post implantation. Evaluation of various preculture strategies, with different cell types, and at large size scales, with eventual in vivo evaluation in large animal models will be necessary to gain a full understanding of the in vitro benchmarks that are necessary for optimal in vivo translation.

### 5.4 Animal models and in vivo evaluation

Finally, while many designs of engineered discs have been described in the literature over the past decade, very few studies have evaluated engineered discs in vivo within the spine. Additionally, most of the in vivo studies to date have been performed in the rat caudal spine, which possesses a very different anatomy and mechanical loading environment than the human spine.^42^, ^54^ While studies in small animal models such as the rat tail have yielded promising results, implantation of tissue‐engineered discs in the rat lumbar spine, and recently in larger animal models has highlighted new challenges that will need to be overcome, such as implant migration out of the disc space and retention of matrix composition within the construct.^50^, ^58^, ^67^

Considering the challenge associated with implant migration, it is probable that some form of fixation will be necessary to stabilize the implanted motion segment and facilitate initial integration of the engineered disc with the native tissue. However, chronic immobilization is contrary to the goal of tissue engineered disc replacement, and is known to have detrimental effects on disc biology and structure.^112^, ^113^ In animal models, provisional fixation could be provided using bioresorbable implant materials, or fixation designs that provide stability but allow for the transmission of axial loads to the engineered disc.^114^, ^115^ Clinically, immobilization may be achieved via external bracing, which in a pilot study successfully prevented extrusion of whole motion segment allografts in the human cervical spine.^116^ Future work will need to establish the optimal duration of immobilization and time point for restoration of physiologic loading following tissue engineered disc implantation.

Additionally, while some work has been performed to understand the mechanical properties of the interfaces between engineered AF and NP tissues in vitro, the strength of these interfaces generally do not match that of the native tissues,^53^, ^67^ and little consideration has been placed thus far on assessing the integration between the engineered disc and native tissue beyond qualitative histology. Functional integration of an engineered disc with the native tissue is a crucial benchmark in the path to clinical translation, and as such, a thorough assessment of the mechanical properties in compression, tension, and torsion of motion segments implanted with engineered discs will be necessary. Implantation of engineered discs in the spines of large animals in long‐term studies will be necessary to achieve this goal and further the translation of engineered disc technology toward clinical use in human patients.

While there is no true consensus on the ideal animal model for intervertebral disc research, the most commonly used large animal models are dogs, sheep, goats, and pigs.^117^ Discs from these species range in height from approximately 3 to 5 mm, with lateral and anterior‐posterior dimensions ranging from approximately 15 to 46 mm.^117^, ^118^, ^119^, ^120^ These dimensions are smaller than the average human lumbar disc size, but more closely approach human cervical disc dimensions.^117^ Motion segment mechanical properties in these species are also similar in many aspects to human, however pig and sheep discs have significantly higher torsional stiffness and significantly lower compressive range of motion compared to human discs.^118^, ^119^ The goat and sheep cervical spines may be particularly useful models for the evaluation of tissue‐engineered discs due to the semi‐upright nature of their neck, particularly considering the cervical spine may be a logical first target for engineered discs in human patients. It is also important to note that in most animal studies tissue engineered discs are implanted into healthy animals with nondegenerative discs. In human patients with disc degeneration, alterations to adjacent spinal structures, such as the EP, facets, and muscles will be present, as well as global comorbidities including diabetes and osteoporosis, which may impact the outcomes of engineered disc replacement.^121^

Currently, selection of the most appropriate animal model is in the hands of the researcher given that, unlike for the cartilage repair field, for example, no guidance documents exist from regulatory agencies, such as the Food and Drug Administration (FDA), to direct translational animal studies in the spine. Of note, although guidance documents exist for the cartilage repair field, studies in this area are poorly adherent to the recommendations that have been set forth.^122^ Moving forward, the spine field should learn from this and strive to be more rigorous in justifying animal model selection and in reporting outcomes from animal model studies. Outcomes of animal studies should ideally encompass both the assessment of structure and function of the engineered disc in vivo. As such, noninvasive imaging modalities such as MRI could be a powerful tool for the longitudinal in vivo evaluation of engineered disc replacements. Additionally, there is a need to develop new grading scales for MRI and histology to assess regeneration of the disc, akin to the ICRS score for cartilage repair,^123^ which will aid in the comparison of results across studies. Finally, these structural and histological outcomes ultimately need to be correlated with pain, as this is the primary reason for surgical interventions in humans with disc disease. Consensus on the appropriate animal model to use for whole disc tissue engineering studies and the minimum set of outcomes to report will be necessary to progress the clinical translation of tissue‐engineered discs.

## 6 CONCLUSIONS AND RECOMMENDATIONS

**Figure 4:**
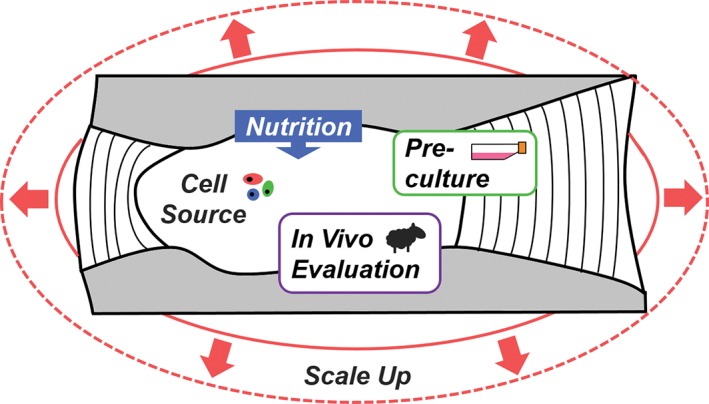
A summary schematic illustrating the challenges facing the field of whole disc tissue engineering

With the pervasive burden of back and neck pain in modern society, there is substantial promise for tissue‐engineered replacement discs to advance the clinical treatment of intervertebral disc degeneration. Although tissue engineering of the intervertebral disc has progressed in the past decade, from design concept to in vivo evaluation in animal models, there are significant challenges yet to be addressed, including construct size, cell source, culture strategies, and translational models (Figure 4). To enhance the rate of clinical translation, we recommend future research efforts should be focused on the following areas:

- Scale‐up of tissue‐engineered discs toward clinically relevant size scales.
- Identification of the optimal cell source of disc tissue engineering.
- Defining benchmarks for success during preculture via the rigorous characterization of the biologic, compositional, structural, and mechanical properties of engineered discs.
- Comprehensive evaluation of engineered discs in vivo in the spine of small and large animal models.
- Development of MRI and histologic grading scales for disc regeneration and correlations to clinically relevant outcomes (including reduction in pain).
